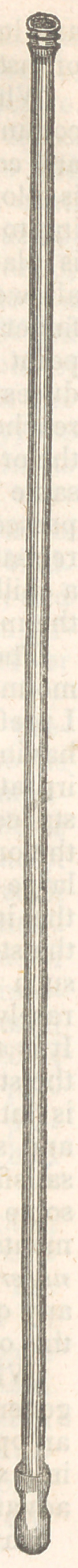# On Strictures of the Rectum

**Published:** 1845-02

**Authors:** Thomas D. Mütter

**Affiliations:** Professor of Surgery in Jefferson Medical College, Philadelphia


### *On Strictures of the Rectum.* By Thomas D. Mütter, M. D., Professor of Surgery in Jefferson Medical College, Philadelphia.

Unquestionably there is no disease more inconvenient, distressing, and in many cases indomitable, than "stricture of the rectum!" Assuming a variety of forms, attacking all ages and all conditions of life, sparing neither the frail nature of woman, nor the sturdy frame of man, the surgeon, in all times and in every land, has regarded the complaint with peculiar and often most painful interest. Fortunately, the disease cannot be considered one of common occurrence; although a few members of the profession, in the base attempt to gain fortune and notoriety, have disgraced both their science and themselves by declaring the contrary, and who endeavour to induce every dyspeptic patient who falls into their hands to fancy himself affected with a stricture, or some dreadful complaint of the rectum. But the experience of the best surgeons, I repeat, declares the disease to be rare.

Strictures of the rectum may be divided, in the first place, into the *Simple* or *Non-malignant*, and the *Malignant*. Under the *first* head are ranged the *Spasmodic* or *Functional*, and the *Permanent* or *Organic;* under the *second*, the *Schirrous*, *Lardaceous*, and *Fungoid Degenerations*.

### OF SIMPLE OR NON-MALIGNANT STRICTURE.

1. *Spasmodic Stricture.*—This form of the disease presents itself, as just stated, under two aspects, the *Spasmodic and Permanent.* The *first* is so rarely met with, that several have doubted its existence, but nearly every surgeon of experience has had occasion, in one or more instances, of verifying the fact of its occasional presence. Much discrepancy of opinion prevails in relation to the precise seat of spasmodic stricture ; some declaring that, like the permanent variety, it occupies the lower portion of the intestine, while others contend that the upper third is most generally affected. The probability is, that every section is liable to this irregular contraction, but from the fact that the lower portions are furnished with larger and more powerful muscular fibres, the stricture more frequently involves these parts, so that it can readily be reached in such cases with the finger.

The causes of this irregular contraction, or spasm of the muscular coat of the rectum, are exceedingly obscure, but it is usually attributed to some vitiation in the secretions of the intestinal canal. This view of the subject is rendered plausible by the great benefit derived, in many cases, from a regulation of the patient's diet, and a course of alterative medicines. Sometimes, however, it appears strictly dependant upon irritation of the rectal nerves, and then the influence of a change of diet is apparently but trifling. Occasionally, too, as in the case reported by Mayo, the constant use of medicine, injections, and the bougie, has, by keeping up continual irritation in the part, been productive of the very disease it was intended to overcome. Hereditary predisposition to affections of the rectum has likewise been cited as one of the causes of the complaint, but apparently with but little authority. Lastly, it is supposed that sex exerts some influence, the complaint being most frequently met with in the female.

The symptoms indicative of spasmodic stricture resemble, in many respects, those which characterize the permanent variety. For example, there is obstinate constipation, small and figured fæces or pellets, sense of weight and pain in the loins, fulness of the lower part of the abdomen, flatulence, in short, all the usual phenomena of dyspepsia are present. Frequently, too, the bladder and urethra are irritable, and the urine turbid and loaded with lithic acid. But when we examine the rectum with the finger and speculum, *there is neither induration, nor thickening of the tunics of the intestine, and if we press firmly against the contraction with the finger or bougie, it will, in most cases, readily yield!* This, taken in connection with the fact, that the dyspeptic symptoms usually *precede* the occurrence of constipation, and the other symptoms of stricture, will enable us to distinguish the nature of the complaint at once.

This form of stricture is liable to be confounded with *neuralgia of the rectum, sacs, the natural folds of the rectum, relaxed rectum, and permanent stricture;* but a careful examination of the case will be sufficient to prevent any error in diagnosis. Of all the varieties of stricture, this is by far the most susceptible of relief, and will yield in most cases without much delay. Occasionally, however, it is more obstinate, and sometimes, from the fact that the causes upon which it depends cannot be removed, it continues for several months, and may finally prove the immediate source of dissolution.

When an opportunity for examining the parts after death is afforded, there is found in most cases scarcely any appreciable lesion of the gut. Sometimes it is a little dilated above the contraction; and where the case is of long standing, there may exist either thickening or atrophy, or ulceration of the different coats, especially the mucous.

In the management of spasmodic stricture we must endeavour to remove, as speedily as possible, the cause upon which the irritation depends. Where a vitiated condition of the alvine secretions appears to be the chief agent, we should regulate the diet, give alterative doses of blue pill, with some bitter and alkaline infusion, employ warm or cold baths, according to circumstances, frictions, moderate exercise, and change of climate. Where spinal irritation seems to be the origin of the difficulty, the usual remedies for this complaint are indicated. And lastly, when we are told that all the remedies calculated to afford relief have, after repeated trials, failed, we may, with advantage in most cases, order a cessation of all medical or surgical treatment, and trust the case to the vis medicatrix naturæ. But in nearly every instance much benefit may be derived from local measures. When, for example, there is present some irritation or inflammation of the rectum, leeches, anodyne injections, hip baths, diluent drinks, and small doses of balsam copaiba, should always be employed. Often, too, I have known the nitrate of silver, applied either in solution or the solid state, quiet the irritation in the course of a few hours. Lastly, the cautious use of a bougie will sometimes prove of great service, but the instrument should never be employed when it produces pain or increases the irritation. The bowels, under all circumstances, should be kept in a loose condition, by emollient injections, if possible, but all active purging, for obvious reasons, must be avoided.

2. *Permanent Stricture.*—Permanent or organic stricture in nearly every case is the result of thickening and condensation of the mucous and submucous cellular coats of the intestine. Usually this thickening takes the form of a ring, "from a third to half an inch or even more in depth, which projects into and narrows the channel!"—(Mayo.) In some cases, however, it is much more extensive, and may involve several inches of the gut. The orifice in the centre of this ring is, for the most part, smooth, and of less thickness than the base. Sometimes, however, instead of the ring, but a portion of the circle of the intestine is involved, and a sort of *shelf* is thus formed on one side. Most surgeons unite in the opinion that permanent stricture is the result of some previous inflammatory action. Any cause, therefore, calculated to produce this condition of the part, may lay the foundation of contractions. Thus, dysenteric inflammation, the frequent use of injections, obstinate constipation, and the consequent passage of hardened fæces, unnatural practices, operations about the anus and rectum, producing, as they do, irritation and inflammation, with deposites of coagulable lymph, may develope a stricture. Sir C. Bell has assigned a different origin for this defect. According to him, "it is a consequence of inflammation in the gut, excited by frequent ineffectual efforts to propel the fæces in a constipated state of the bowels. The sphincter, in this condition, does not relax, nor does the intestine itself act. The whole propelling power is in the abdominal muscles. The rectum, urged down by pressure from above, *forms a fold of the inner coat,* just above the inner sphincter. By repetition, inflammation and adhesion of the outer surfaces of the fold take place, and by these means losing its softness, and yielding nature, it becomes a permanent septum, extending nearly across the gut."

That such a cause may produce stricture is sufficiently obvious; but the experience of nearly all goes to prove, that the disease much more frequently originates in simple deposites of lymph in the mucous membrane, or the cellular tissue beneath it.

The ordinary seat of permanent stricture is about two and a half to four inches above the orifice of the intestine. Occasionally it is detected much higher up, say six or seven inches, but this is by no means a common event, as a few would have us believe, and when met with, always demands a more difficult and careful treatment than when it occurs lower down. The strictures, sometimes detected in the *colon*, should not, I conceive, be considered under the head of strictures of the *rectum*, as is done by some. Their presence cannot be detected prior to death, at least in the majority of cases; and when the diagnosis is made out during the life of the individual, it is productive of no practical benefit whatever.

In most cases the disease is slowly developed, and the symptoms indicative of its presence are, consequently, gradual in their approach. Occasionally, however, as Mr. Colles has well observed, the patient appears to be fully aware of the moment of the first attack, "for he will tell us that, without any previous illness, the bowels, at a certain period, *suddenly* became costive; that, for the purpose of relieving them, he took large and repeated doses of physic, for three, four, or five successive days; that at length his bowels *suddenly* gave way, and a very severe purging took place, which, having continued for a day or two, was then succeeded by those symptoms which attend the disease when fully formed!"

The symptoms indicative of stricture vary somewhat in different cases, but in nearly all there exists constipation of the bowels, occasionally alternating with looseness; a sense of weight and uneasiness, with spasmodic pain in the lower part of the abdomen, distention of the intestines with flatus, eructation, and uncertain appetite. As the disease progresses the constipation becomes more obstinate, and the quantity of fæces voided at each effort is small, moulded into narrow strips like ribbon, or formed into round and hard pellets. It is well to bear in mind, however, that fæces of a natural size and shape are occasionally found, even where the stricture is very extensive. This is owing to the circumstance first pointed out by White, that as the disease advances, the inferior portion of the intestine loses its power; and as the quantity of fæces passed at a time through the stricture is not sufficient to stimulate this partially paralysed portion to contraction, an accumulation takes place *below the stricture,* and then, when a sufficient amount is collected to excite the rectum to an expulsive effort, the mass discharged presents the natural size and form.

As the patient is unable to evacuate the bowel completely at once, he is obliged to repeat the attempt several times a day, and the constant straining against the stricture causes the part to inflame, and sometimes to ulcerate. There is then excessive pain after or during an evacuation, accompanied often by a discharge of thin, brownish mucus, or pus and blood. The occurrence of pain with the discharge of mucus always indicates inflammation, inasmuch as the stricture itself gives rise rather to a source of uneasiness than positive pain. If the inflammation extends further, abscesses are developed in the surrounding cellular tissue, which ultimately discharge into the neighbouring cavities. The orifice of the rectum, in many cases, presents a peculiar swollen and livid appearance, in consequence of the engorgement to which the hemorrhoidal vessels are subjected by the repeated efforts at evacuation of the bowels, and there is also a most troublesome itching or burning about the anus. Frequently the irritation extends itself to the adjacent organs, giving rise to congestion of the womb, affections of the bladder and urethra, and neuralgic pains down the thighs and legs.

The general health of the patient, in the commencement of the complaint but slightly affected, sooner or later becomes impaired, his appetite fails, his digestion is bad, his temper irritable or desponding, his strength diminishes, emaciation speedily sets in, and unless he is relieved by proper treatment, death soon closes the scene. Occasionally, however, he drags on a miserable existence for many years, and in some rare cases the general health may be but slightly impaired, and the patient remain totally unconscious of the existence of stricture.

But these symptoms may be developed by other diseases of the rectum; and in order to ascertain positively the nature of the complaint, it becomes necessary for us to examine the part more closely. By far the best instrument for this purpose, at least in the majority of cases, is the finger, with which the stricture can readily be felt. The patient should be placed on his side, with the thighs flexed upon the pelvis, the legs upon the thighs, and the buttocks brought near the edge of the bed. The surgeon then introduces his finger, previously well oiled, and carries it carefully and slowly up to the seat of the disease, and then gently endeavours to discover the nature of the obstruction, and its extent. Unless the parts are inflamed, this operation, when properly performed, gives rise to no pain whatever. Some recommend for the examination of the rectum a speculum, but I have never been able, myself, to derive the slightest assistance from its use; and as it requires an unnecessary exposure of the person of the patient, and the operation is more or less painful, I have for some time abandoned its employment in such cases.

When the stricture is situated high up, and beyond the reach of the finger, we are obliged to employ either a common bougie, or the rectum sound of Sir C. Bell, which consists of an ivory ball mounted on a stalk of whalebone. This instrument, from its flexibility, and from the fact that after the ball is once introduced, the anus is freed from all distention, is by far the most useful that we possess, and with it we can readily ascertain the location, extent, and even consistence of the stricture; and also the presence of a second, should another exist. This examination, unless the parts are inflamed, should give rise to little or no pain, nor is it attended with the slightest hazard, provided it be carefully performed. The case of perforation of the intestine with the bougie, reported by Mr. Crosse, of Norwich, to Mr. Mayo, proves, however, that unless the operation is performed with gentleness, a fatal result may be the consequence.

From the fact that several other affections of the rectum, as well of the adjacent organs, may give rise to nearly all the prominent symptoms of stricture, we find that errors in diagnosis are not unfrequently committed. For example, *retroversion of the uterus*, *enlargement of this organ*, *stone in the bladder*, *enlarged prostate*, *tumours of different kinds developed in the vicinity*, *relaxed rectum*, *prolapsus ani*, *piles*, *and retained fæces*, (which sometimes push before them a fold of mucous membrane,) by diminishing the caliber of the rectum, and thus giving rise to certain symptoms that belong, for the most part, to stricture, have all been mistaken for the disease in question. Chronic inflammation of the vagina, and ulcers, both simple and malignant, of the rectum itself, have been confounded with stricture. There is likewise a singular affection of the rectum, described by Sir Benjamin Brodie, in which the gut is studded with *small tumours*, that has been mistaken for stricture. But a careful examination with the finger or bougie, will enable us in all cases to arrive at a correct diagnosis; and every malignant affection is readily distinguished by the severe pain and great constitutional disturbance which usually indicate its presence.

Stricture of the rectum is generally a tedious complaint; and even after we succeed in removing it, there always exists a disposition in the parts to a reproduction of the difficulty. When it consists of a simple thickening of the coats of the intestine, without any malignant tendency, and when it is of recent formation, it is for the most part readily managed; but if the case is neglected until the constitution begins to fail, and adjacent organs become involved in the disease, all the resources of our art will often prove either nugatory or positively injurious. Occasionally, too, the patient is carried off suddenly, with all the symptoms of strangulated hernia, in consequence of a piece of hardened fæces blocking up completely the orifice of the stricture. The appearances on dissection vary somewhat in different cases. When the disease is the result of previous inflammation, we find at the seat of stricture, the mucous membrane, the cellular coat, and sometimes even the muscular, thicker and harder than usual. Above the stricture the intestine is usually dilated and thinned, in consequence of the accumulation of fœcal matter; below it the gut seems to be relaxed, and softer than natural. When the stricture has been formed by a pushing down of a fold of mucous membrane, we find this fold firm and hard from the deposite of coagulable lymph. Sometimes, in each variety, we have ulceration of the stricture and mucous membrane in its vicinity.

Lastly, when the disease is of long standing, the intestine is usually incorporated with the vagina in the female, and the bladder in the male, or with the fold of another intestine above; and when ulceration has taken place, we find fistulæ forming in different directions, some communicating with the bladder or vagina, others running into the cellular tissue of the pelvis.

After, by careful examination, we have detected the presence of a stricture, our attention is next to be directed to its seat, its complications and condition. If low down, uncomplicated with disease of other organs, and characterised by little or no irritability, we may commence its treatment immediately. On the other hand, if very high up, so as to be beyond the reach of the finger, it becomes a question whether it should be interfered with or not. For my own part, I have never found much advantage from the use of instruments in such cases; but inasmuch as others declare that relief has been derived from a well directed treatment, the attempt may be made; but should it produce much irritation, no prudent surgeon, I think, would persevere in its continued employment. In such a stricture a palliative treatment is all that, in most cases, can with safety be had recourse to.

Again, when there exists some disease of neighbouring organs, this must be removed, if possible, before we commence the treatment of the stricture; but when, from the peculiarities of the case, this cannot be accomplished, we should endeavour to manage both at the same time, or carefully ward off the dangers of the former until we have succeeded in relieving the latter. Lastly, when the stricture is irritated or inflamed, or the rectum itself either above or below it, is in a similar condition, no attempt upon the contraction should be commenced until, by previous emollient and anodyne injections, hip baths, mild diet, rest, and leeches to the anus, the unfavourable condition for the use of instruments is entirely removed.

When the parts are in a proper condition, the treatment should commence with the introduction of a bougie, of suitable size and construction, through and through the stricture, in which it is allowed to remain for ten, twenty, or thirty minutes, according to circumstances. To introduce the instrument the patient is placed either on his side, near the edge of the bed, or is allowed to kneel on a chair. The surgeon next carries his fore finger, previously well oiled, up to the stricture, and places its point in the orifice, then taking the bougie, he carefully introduces it along the finger, which serves as a guide, until he reaches the seat of disease, and having lodged its extremity in the orifice of the contraction, he withdraws the finger, and at the same time pushes up *slowly* and *gently* the bougie, until it has passed through the resistance. The operation may be daily repeated should it develope no irritation, but if it is followed by a chill or symptomatic fever, there will be hazard in repeating the measure so frequently.

The bougies I employ are made either of metal or wood, and mounted upon a flexible stem, as recommended by Dr. Bushe. I prefer these to the gum elastic or wax, in consequence of my having seen in stricture, of both the urethra and rectum, much irritation occasioned by bougies made of the last named substances; and by having them mounted, we avoid distention of the orifice of the rectum, which is sometimes so painful when a large bougie is used that it becomes insupportable. The size of the instruments should, of course, be gradually increased, until the stricture is *sufficiently dilated*, by which I mean, opened to such a degree that the fæces pass readily. The intestine is rarely, if ever, restored to its original calibre, nor is this essential. It is always proper, when there is any lodgement of fæces below the stricture, to wash them out with an enema before the bougie is introduced. When thus treated the stricture *slowly* yields, and several weeks or even months may elapse before we are satisfied that we have gone far enough. Anxious to save time, some surgeons have preferred a more rapid dilatation by instruments of various kinds; but the golden rule in this complaint is, *never to be in a hurry!* and I have therefore never employed any other agent to assist the ordinary bougie than the knife, and this only in certain cases.

When the introduction of the instrument causes pain, I have generally administered, about an hour before the operation, either an opiate suppository, or an injection of fifty drops of tinct. opii in a small quantity of water. Sometimes, too, I have derived advantage from the use of a warm emollient injection, just before the introduction of the bougie. But occasionally it happens, that with all our care the operation is excessively painful; in such cases I employ a bougie of a construction essentially different from those hitherto described. It consists of a small bladder tied firmly to a nozle furnished with a stop-cock and screw nipple, to which is attached a flexible tube, twelve or fifteen inches in length. When about to be introduced the bladder is moistened with warm water and then oiled, and the tube screwed on. A blunt probe is then engaged in a fold of the bladder near its apex, and the two carried along the fore finger, previously introduced as a guide, until the orifice of the stricture is reached. A very slight effort will carry the probe and bladder through the stricture, which being accomplished, the probe is withdrawn, and then the surgeon, applying his mouth to the tube, *inflates the bladder until the patient complains of pain;* the stop cock is then turned, the tube unscrewed, and the "*air bougie*" allowed to remain as long as the patient can bear it. I have used this instrument for several years, and with great advantage, and am not aware that it has ever been employed by any one else. The nearest approach to it was in the instrument of Calvert, who proposed to introduce a *prepared gut, and then fill it with water,* as is done by Mr. Arnott in some of his various dilators. Whether he used this instrument or not I am unable to say.

To remove the *air bougie* it is necessary to turn the stop-cock, which allows the air to escape, and then the bladder can be withdrawn without the slightest difficulty.

When the stricture is near the orifice of the anus, of small extent, and uncomplicated with other diseases, Wiseman long since proposed its division with the knife. Many modern surgeons of high authority, and among them Sir B. Brodie, have adopted his views, and recommend the same procedure. That the operation is productive of decided benefit in some cases, no one who has ever had occasion to put it in practice will doubt; but it should be recollected that, unless carefully performed, severe and even fatal hemorrhage, or intense inflammation may ensue. To obviate these dangers the incisions must not be extended more than a line or two, and should be made in three or four places. The best mode of performing the operation is, after having first carefully washed out the rectum, to dilate the the anus with a speculum, so as to obtain a view of the stricture, or when this cannot be done, to introduce the finger and pull down the obstruction as advised by Dr. Bushe; and then, with a hernia knife, or probe-pointed bistoury, wrapped to within half an inch of its point, or a bistoury caché, divide the margin of the stricture as directed. To prevent union of the incisions, we should at once introduce through the stricture a *short bougie* of wax furnished with a loop of tape. The instrument must be carried completely within the anus, so as to prevent irritation of the sphincter, and then attached by the tape to an ordinary T bandage.

Usually the operation is painful, and it will be proper to administer a full dose of some anodyne, after the patient is placed in bed. The bougie should be allowed to remain at least twenty-four or thirty-six hours, provided it produces no irritation; at the expiration of this period it must be withdrawn, the bowels opened with an injection, and then, as soon as the irritation of the evacuation is over, the instrument may be replaced and worn as long as the patient can bear it. After a few days we treat the case as we would one in which the knife had not been employed. When the bougie causes pain and inflammation it must be removed at once, and not again introduced until the parts are in a less irritable condition.

During the management of this form of stricture the patient must be confined as much as possible to the horizontal position, in order to diminish the risk of inflammation from the different operations; have his bowels kept in a proper condition by emollient injections, or the *mildest laxatives*, as the confection of senna, &c.; live upon a mild though nutritious diet; use anodyne injections or suppositories, and hip-baths if there is much pain about the part; and finally, should inflammation supervene, it must be treated by leeches, diluent drinks, rest, anodynes, &c.

Under the impression that benefit might be derived from the various preparations usually considered as promotive of absorption, it has been recommended to smear the bougie with mercurial, iodine, iodide of potassium or lead, or belladonna ointment; and I think I have seen the practice followed by good results.

There are a few troublesome complications with which we have to contend in nearly every case, and we must, therefore, be prepared to meet them. One of the most inconvenient is fæcal retention, large masses of fæces being occasionally lodged above the stricture, and if neglected, often give rise to symptoms of strangulated hernia, and may cause the patient's death. To relieve this difficulty, we must pass a gum elastic catheter, with large eyes, through the stricture, and then throw up with a syringe a sufficient quantity of warm soap and water to render the fæces liquid. But it may happen that the fæcal mass is so solid that little or no impression is made upon it by the water; where such is the case, we must employ the scoop along with the injection.

Another troublesome and often incurable complication is the establishment of a fistulous connection between the neighbouring cavities or surfaces and the rectum. In all these cases it is our duty, first, to remove the stricture, if possible, and then treat the fistula. When we have the rectum and bladder communicating, we must endeavour to keep a catheter in the bladder, and occasionally touch the orifice of the fistula with the lunar caustic. When the fistula is recto-vaginal, the caustic, cauterising, or some of the various operations devised for this defect, must be had recourse to. Lastly, when the fistula terminates near the anus, or upon the buttock, it may be treated in the manner usually employed for fistula located in these spots.

When ulceration takes place we are generally, though not invariably, obliged to suspend the use of the bougie, such is the suffering produced by its introduction. In these cases we must endeavour, by emollient injections, rest, moderate diet, and the use of the *nitrate of silver*, to cause the ulcer to heal. Often the most soothing applications here are, injections of a little warm castor oil, or equal parts of linseed oil and lime water, or fresh lard, or carrot water, or flaxseed mucilage.

The occurrence of diarrhœa is another exceedingly unpleasant attendant upon stricture; but, for the most part, it will yield to the administration of the usual remedies for this complaint.

The irritation of the bladder, or vagina, or womb, whenever they supervene, must, of course, be treated on general principles. But in spite of all our care, it may so happen that the stricture continues to increase, until at length complete obstruction takes place; or, instead of this, we may be called to a patient who has never been subjected to treatment and find him in the same condition, unable to pass his fæces at all, and hence in hourly danger of death. In such cases one of two things must be done—either the stricture is to be *forced*, or an *artificial anus established in the lumbar region.* If we have reason to suppose that the stricture is *not of great extent*, if it be *low down*, and if the *rectum is in a sound condition*, we should perforate the obstruction with a curved trocar, and then dilate the wound with a bistoury caché; after which bougies must be employed, until the orifice is sufficiently large. On the other hand, when the stricture is *extensive*, *situated high up*, and the *intestine appears diseased*, the *artificial anus* may be established, although it is a question whether such an alternative is not worse even than death.

Finally, it must be borne in mind that these causes of simple stricture, though often *readily relieved*, are *rarely, if ever, cured;* that is, the patient will be obliged, as long as he lives, to employ occasionally the bougie, regulate his diet, avoid constipation, and remain as much at rest, in the horizontal position, as possible.

(*To be continued.*)

## Figures and Tables

**Figure f1:**
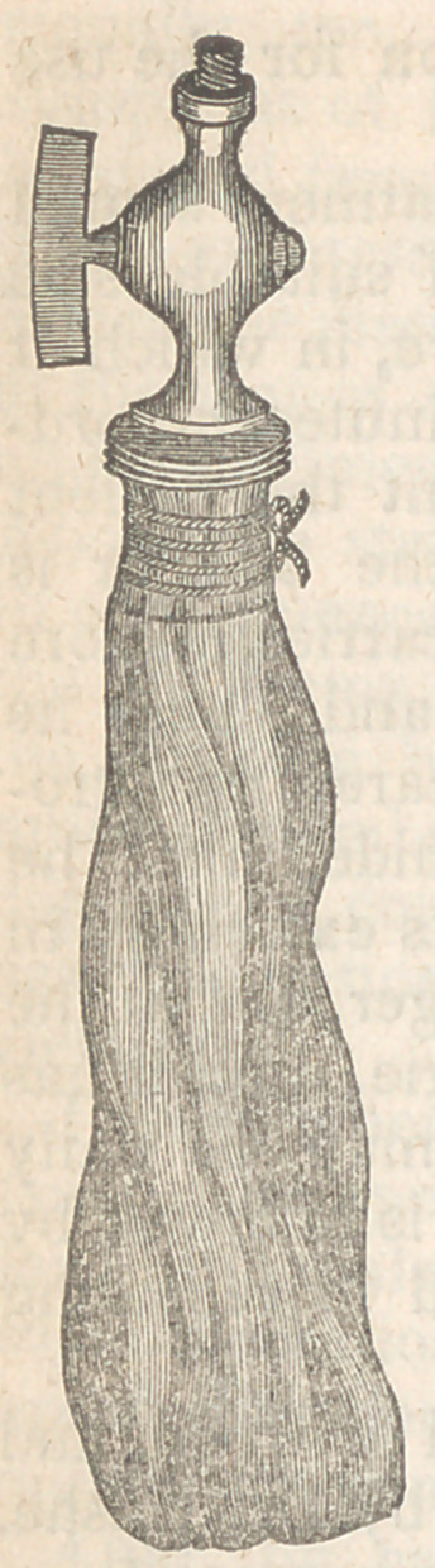


**Figure f2:**